# Polycyclic aromatic hydrocarbon-substituted push–pull chromophores: an investigation of optoelectronic and nonlinear optical properties using experimental and theoretical approaches

**DOI:** 10.3906/kim-2102-22

**Published:** 2021-10-19

**Authors:** Çağatay DENGİZ

**Affiliations:** 1 Department of Chemistry, Middle East Technical University, Ankara Turkey

**Keywords:** polycyclic aromatic hydrocarbon, chromophore, charge-transfer, push–pull, [2+2] cycloaddition-retroelectrocyclization

## Abstract

A series of new push–pull chromophores were synthesized in moderate to very high yields (65%–97%) by treating TCNE and TCNQ with alkynes substituted by electron-rich diethylaniline and polycyclic aromatic hydrocarbons. Some of the chromophores exhibit strong intramolecular charge-transfer bands in the near-IR region with λ_max_ values between 695 and 749 nm. With the help of experimental and theoretical analysis, it is concluded that the trend in* λ*_max _values is affected by PAH substituents sterically, not electronically. Steric constraints led to the increased dihedral angles, reducing conjugation efficiencies. The absorption properties of push-pull compounds have been investigated in solvents possessing different polarities. All chromophores exhibited positive solvatochromism. As an additional proof of efficient charge-transfer in push–pull chromophores, quinoid character (d*r*) values were predicted using calculated bond lengths. Remarkably, substantial d*r* values (0.045–0.049) were predicted for donor diethylaniline rings in all compounds. The effects of various polycyclic aromatic hydrocarbons on optical and nonlinear optical properties were also studied by computational methods. Several parameters, such as band gaps, Mulliken electronegativity, chemical hardness and softness, dipole moments, average polarizability, first hyperpolarizability, were predicted for chromophores at the B3LYP/6-31++G(d,p) level of theory. The predicted first hyperpolarizability β_(tot) _values vary between 198 to 538 × 10^–30 ^esu for the reported push–pull chromophores in this study. The highest predicted β_(tot) _value in this study is 537.842 × 10^–30 ^esu, 8150 times larger than the predicted β_(tot) _value of benchmark NLO material urea, suggests possible utilization of these chromophores in NLO devices. The charge-transfer character of the synthesized structures was further confirmed by HOMO-LUMO depictions and electrostatic potential maps.

## 1. Introduction

Donor-π-acceptor (D-π-A)-type push–pull chromophores, are well-known for their desirable features, such as tunable strong intramolecular charge-transfer bands that absorb light in a wide range of area including visible and near-IR regions, spectacular nonlinear optical properties, excellent solubility, and high thermal stabilities [1–3]. With these desired properties, push-pull chromophores have already been employed in a series of advanced applications such as photovoltaics, [4,5] light-emitting diodes, [6,7] sensors, [1,8,9] and NLO devices [1,10,11]. The successful integration of push–pull systems in high technology areas makes it necessary to design and synthesize new molecular structures with enhanced optoelectronic properties. However, synthetic strategies to access these entities are arguably limited and require multi-step protocols. The formal [2+2] cycloaddition-retroelectrocyclization (CA-RE) is one of the promising reaction candidates to circumvent these synthetic problems [1]. With its high-yielding nature and broad substrate scope, [2+2] CA-RE transformation occurs under very mild conditions without requiring a catalyst and fulfills all the requirements to be referred as a “click-type reaction” [12,**13**] [2+2] CA-RE transformation requires electron-rich alkynes and electron-deficient alkenes to synthesize nonplanar D-π-A systems [1]. Bruce and co-workers reported the first example of [2+2] CA-RE reactions between ruthenium-substituted acetylides and tetracyanoethylene (TCNE) in 1981 [**14**]. Later, metal-free substrates have also been employed in CA-RE reactions for the synthesis of structurally demanding push-pull chromophores by Diederich and co-workers [11]. The short and easy-to-perform CA-RE method has increased the variety in the design of the target push–pull chromophores. When the structures of push-pull chromophores obtained by CA-RE reactions are examined in detail, derivatization is mostly made in two main parts (donor groups and acceptor groups). The typical electron donors used in CA-RE can be listed as azulenes, [**15**–**17**] tetrathiafulvalenes, [**18**] methoxy groups, [19] dialkyl amines [20], ferrocenes, [21] triazenes, [22] thiophenes, [19] ureas, [8] heteroazulenes, [23] ynamines [24], and ynamides [24]. As mentioned earlier, another way to diversify the products obtained by click-type [2+2] CA-RE reactions is to change acceptor units. Similar to the donor groups, successful utilization of many acceptor units such as TCNE [21,25], 7,7,8,8-tetracyanoquinodimethane (TCNQ), [26] 6,6-dicyanopentafulvenes (DCFs),[27] N,N’-dicyanoquinonediimides (DCNQIs), [28] and 2-(dicyanomethylene)indan-1,3-dione (DCID)[29] have been demonstrated in the literature [1]. Apart from studies on donor and acceptor groups, a significant amount of research has also been conducted on π-linkers [1,20,21]. More recently, Diederich and co-workers reported Aviram–Ratner-type dyads with rigid σ-linkers obtained by CA-RE cascade [30]. Due to the nature of the [2+2] CA-RE transformations, substituents have often been used to improve solubility and stability, to increase the electron-acceptor properties, or to incorporate a second donor group into the structure. Trolez and co-workers recently reported ynamide-based push–pull systems with pyrene and perylene substituent units as near-infrared emitters [25]. Another study from the Diederich Group involved the synthesis of corannulene-based push-pull chromophores [31]. Historically, polycyclic aromatic hydrocarbons (PAHs) have been key building blocks for constructing complex π-conjugated systems [32]. Among various PAH molecules, complex push–pull systems having core PAH structures have always been very attractive targets [1,33–36]. Synthetic challenges and characterization issues associated with the solubility of the polycyclic aromatic hydrocarbons are the main barriers complicating the research into these structurally exciting molecules. In this study, we present a detailed systematic survey on structure-property relationships of naphthalene and phenanthrene-substituted push–pull chromophores. The flexibility of the CA-RE reactions allowed us to use both TCNQ and TCNE at the final stage of the synthesis to implement cyano-based acceptor groups. Optoelectronic properties of the synthesized push-pull chromophores have been investigated both experimentally (UV/vis, solvatochromism studies) and theoretically (TD-DFT studies, NLO analysis, HOMO-LUMO visualizations, electrostatic potential maps).

## 2. Experimental section

### 2.1. General information

All reagents were purchased as reagent grade and used without further purification. Compounds 2 [37], 3 [37], 6 [38], 7 [38], 10 [39], and 11 [39] were prepared according to literature procedures. Solvents for extraction or column chromatography were distilled before usage. Reactions under exclusion of air or water were performed in oven-dried glassware and under argon or N_2 _atmosphere. Column chromatography (CC) was carried out using SiO_2_-60 mesh. Analytical thin-layer chromatography (TLC) was performed on aluminum sheets or glass plates coated with 0.2 mm silica gel 60 F254; visualization with a UV lamp (254 or 366 nm). Evaporation in vacuo was performed at 25–60 °C and 900–10 mbar. Reported yields refer to spectroscopically and chromatographically pure compounds that were dried under a high vacuum (0.1–0.05 mbar) before analytical characterization. ^1^H and **^13^** C nuclear magnetic resonance (NMR) spectra were recorded at 400 MHz (^1^H) and 100 MHz (**^13^** C), respectively. Chemical shifts *d* are reported in ppm downfield from tetramethylsilane using the residual deuterated solvent signal as an internal reference (CDCl_3_: d_H_ = 7.26 ppm, d_C_ = 77.0 ppm). For ^1^H NMR, coupling constants *J* are given in Hz, and the resonance multiplicity is described as s (singlet), d (doublet), t (triplet), q (quartet), and m (multiplet). All spectra were recorded at 298 K. High-resolution mass spectrometry (HR-MS) was performed by the MS-service of the Central Laboratory at Middle East Technical University, Turkey. Masses are reported in m/z units as the molecule ion as [M + H]^+^.

### 2.2. General procedure A: synthesis of TMS-protected alkynes 2, 6, and 10

In a 25 mL round bottom flask aryl bromide (1.00 mmol, 1 equiv.), bis (triphenylphosphine) palladium (II) chloride (0.09 mmol, 0.09 equiv.) and copper iodide (0.09 mmol, 0.09 equiv.) were added. The flask was flushed with nitrogen for 30 min, toluene (6 mL) and diisopropylamine (3 mL) were added via syringe into the flask and flushed with nitrogen for an additional **15** min, followed by the addition of trimethylsilylacetylene (3.00 mmol, 3 equiv.). After stirring overnight at 60 °C, the solvents were removed under reduced pressure, target TMS-protected alkynes 2, 6, and 10 were isolated in 78%–92% yields by performing column chromatography (CC) (SiO_2_; *c*-hexane). 

**Compound 2: **colorless oil; **17**5mg, 78%; CC: (SiO_2_; *c*-hexane); R*_f_* = 0.33 (SiO_2_; *c*-hexane); ^1^H NMR (400 MHz, CDCl_3_, 298 K): δ = 0.29 (s, 9 H), 7.44–7.54 (m, 3 H), 7.74–7.84 (m, 3 H), 8.00 ppm (s, 1 H); **^13^** C NMR (100 MHz, CDCl_3_, 298 K): δ = **13**3.02, **13**2.99, **13**2.1, 128.7, 128.0, 127.92, 127.87, 126.8, 126.6, 120.5, 105.6, 94.7, 0.**17** ppm. Spectral data was consistent with literature [37].

**Compound 6: **colorless oil; 202 mg, 90%; CC: (SiO_2_; *c*-hexane); R*_f_* = 0.40 (SiO_2_; *c*-hexane); ^1^H NMR (400 MHz, CDCl_3_, 298 K): δ = 0.33 (s, 9 H), 7.41 (t, *J* = 7.7 Hz, 1 H), 7.52 (t, *J* = 7.5 Hz, 1 H), 7.58 (t, *J* = 7.6 Hz, 1 H), 7.70 (d, *J* = 7.1 Hz, 1 H), 7.83 (t, *J* = 7.5 Hz, 2 H), 8.33 ppm (d, *J* = 8.2 Hz, 1 H); **^13^** C NMR (100 MHz, CDCl_3_, 298 K): δ = **13**3.5, **13**3.2, **13**1.0, 129.1, 128.4, 127.0, 126.5, 126.3, 125.3, 120.9, 103.2, 99.6, 0.26 ppm. Spectral data was consistent with literature [38].

**Compound 10: **pale yellow oil; 253 mg, 92%; CC: (SiO_2_; *c*-hexane); R*_f_* = 0.37 (SiO_2_; *c*-hexane); ^1^H NMR (400 MHz, CDCl_3_, 298 K): δ = 0.41 (s, 9 H), 7.56–7.63 (m, 1 H), 7.64–7.77 (m, 3 H), 7.85 (d, *J* = 7.9 Hz, 1 H), 8.06 (s, 1 H), 8.44–8.54 (m, 1 H), 8.60–8.74 ppm (m, 2 H); **^13^** C NMR (100 MHz, CDCl_3_, 298 K): δ = **13**2.6, **13**1.24, **13**1.22, **13**0.5, **13**0.1, 128.7, 127.7, 127.21, 127.**17**, 127.08, 127.05, 122.9, 122.7, 119.6, 103.4, 99.3, 0.28 ppm. Spectral data was consistent with literature [39].

### 2.3. General procedure B: synthesis of terminal alkynes 3, 7, and 11 via TMS-deprotection

TMS-protected alkynes (1.00 mmol, 1 equiv.) were dissolved in methanol (10 mL) and THF (10 mL) mixture. Then, potassium carbonate (5.00 mmol, 5 equiv.) was added to this solution. After filtration, evaporation, and column chromatography (CC) (SiO_2_; *c*-hexanes), terminal alkynes 3, 7, and 11 were obtained in 73%–95% yields.

**Compound 3: **grey solid; **13**1 mg, 86%; CC: (SiO_2_; *c*-hexane); R*_f_* = 0.59 (SiO_2_; DCM/*c*-hexane 1:4); ^1^H NMR (400 MHz, CDCl_3_, 298 K): δ = 3.**16** (s, 1 H), 7.45–7.60 (m, 3 H), 7.75–7.90 (m, 3 H), 8.04 ppm (s, 1 H); **^13^** C NMR (100 MHz, CDCl_3_, 298 K): δ = **13**3.1, **13**2.9, **13**2.4, 128.7, 128.2, 127.91, 127.90, 127.0, 126.8, 119.5, 84.1, 77.6 ppm. Spectral data was consistent with literature [37].

**Compound 7: **pale yellow solid; 111 mg, 73%; CC: (SiO_2_; *c*-hexane); R*_f_* = 0.55 (SiO_2_; DCM/*c*-hexane 1:4); ^1^H NMR (400 MHz, CDCl_3_, 298 K): δ = 3.49 (s, 1 H), 7.44 (t, *J* = 7.7 Hz, 1 H), 7.54 (t, *J* = 6.9 Hz, 1 H), 7.60 (t, *J* = 6.9 Hz, 1 H), 7.75 (d, *J* = 7.1 Hz, 1 H), 7.87 (d, *J* = 8.3 Hz, 2 H), 8.37 ppm (d, *J* = 8.3 Hz, 1 H); **^13^** C NMR (100 MHz, CDCl_3_, 298 K): δ = **13**3.6, **13**3.2, **13**1.4, 129.4, 128.4, 127.1, 126.6, 126.2, 125.2, 119.9, 82.1, 81.9 ppm. Spectral data was consistent with literature [38].

**Compound 11: **grey solid; 192 mg, 95%; CC: (SiO_2_; *c*-hexane); R*_f_* = 0.56 (SiO_2_; DCM/*c*-hexane 1:4);^ 1^H NMR (400 MHz, CDCl_3_, 298 K): δ = 3.48 (s, 1 H), 7.58–7.64 (m, 1 H), 7.65–7.74 (m, 3 H), 7.86 (d, *J* = 7.9 Hz, 1 H), 8.07 (s, 1 H), 8.42–8.52 (m, 1 H), 8.64–8.74 ppm (m, 2 H); **^13^** C NMR (100 MHz, CDCl_3_, 298 K): δ = **13**2.8, **13**1.0, **13**0.9, **13**0.4, 129.9, 128.5, 127.6, 127.0, 126.9, 126.7, 122.7, 122.5, 1**18**.4, 81.8, 81.5 ppm (**15** out of **16** signals expected). Spectral data was consistent with literature [39].

### 2.4. General procedure C: synthesis of diethylaniline-substituted alkynes 4, 8, and 12

In a 25 mL round bottom flask N,N-diethyl-4-iodoaniline (1.00 mmol, 1 equiv.), bis (triphenylphosphine) palladium (II) chloride (0.09 mmol, 0.09 equiv.) and copper iodide (0.09 mmol, 0.09 equiv.) were added. The flask was flushed with nitrogen for 30 min, toluene (6 mL) and diisopropylamine (3 mL) were added via syringe into the flask and flushed with nitrogen for an additional **15** min, followed by the addition of terminal alkyne (2.00 mmol, 2 equiv.). After stirring overnight at 60 °C, the solvents were removed under reduced pressure, target alkynes 4, 8, and 12 were isolated in 64%–90% yields by performing column chromatography (CC) (SiO_2_; DCM/*c*-hexane 1:4). 

**Compound 4: **pale yellow solid; 2**13** mg, 71%; CC: (SiO_2_; DCM/*c*-hexane 1:4); m.p. **13**3–**13**5 °C; R*_f_* = 0.28 (SiO_2_; DCM/*c*-hexane 1:4); ^1^H NMR (400 MHz, CDCl_3_, 298 K): δ = 1.19 (t, *J* = 7.1 Hz, 6 H), 3.39 (q, *J* = 7.1 Hz, 4 H), 6.64 (d, *J* = 8.8 Hz, 2 H), 7.40–7.52 (m, 4 H), 7.57 (dd,* J* = 8.4, 1.3 Hz, 1 H), 7.75–7.85 (m, 3 H), 8.01 ppm (s, 1 H); **^13^** C NMR (100 MHz, CDCl_3_, 298 K): δ = **14**7.7, **13**3.3, **13**3.2, **13**2.5, **13**0.7, 128.7, 127.92, 127.85, 127.8, 126.5, 126.3, 121.8, 111.3, 109.0, 91.5, 87.7, 44.5, 12.7 ppm; HRMS: m*/z *calcd for C_22_H_22_N^+^: 300.**17**52; found: 300.**17**54 [M + H]+.

**Compound 8: **pale yellow solid; 270 mg, 90%; CC: (SiO_2_; DCM/*c*-hexane 1:4); m.p. 81–83 °C; R*_f_* = 0.42 (SiO_2_; DCM/*c*-hexane 1:4); ^1^H NMR (400 MHz, CDCl_3_, 298 K): δ = 1.20 (t, *J* = 7.1 Hz, 6 H), 3.40 (q, *J* = 7.1 Hz, 4 H), 6.67 (d, *J* = 8.7 Hz, 2 H), 7.45 (t,* J* = 7.7 Hz, 1 H), 7.48–7.56 (m, 3 H), 7.59 (t,* J* = 7.6 Hz, 1 H), 7.72 (d,* J* = 7.1 Hz, 1 H), 7.79 (d,* J* = 7.1 Hz, 1 H), 7.86 (d,* J* = 8.1 Hz, 1 H), 8.48 ppm (d,* J* = 8.3 Hz, 1 H); **^13^** C NMR (100 MHz, CDCl_3_, 298 K): δ = **14**7.8, **13**3.39, **13**3.37, **13**3.2, 129.7, 128.3, 127.9, 126.62, 126.57, 126.4, 125.5, 122.1, 111.4, 109.1, 96.1, 85.4, 44.5, 12.7 ppm; HRMS: m*/z *calcd for C_22_H_22_N^+^: 300.**17**52; found: 300.**17**44 [M + H]+.

**Compound 12: **pale yellow solid; 225 mg, 64%; CC: (SiO_2_; DCM/*c*-hexane 1:4); m.p. **13**8–**14**0 °C; R*_f_* = 0.25 (SiO_2_; DCM/*c*-hexane 1:4); ^1^H NMR (400 MHz, CDCl_3_, 298 K): δ = 1.21 (t, *J* = 7.1 Hz, 6 H), 3.41 (q, *J* = 7.1 Hz, 4 H), 6.68 (d, *J* = 8.9 Hz, 2 H), 7.54 (d, *J* = 8.9 Hz, 2 H), 7.56–7.74 (m, 4 H), 7.80–7.90 (m, 1 H), 8.05 (s, 1 H), 8.55–8.75 ppm (m, 3 H); **^13^** C NMR (100 MHz, CDCl_3_, 298 K): δ = **14**7.8, **13**3.2, **13**1.7, **13**1.5, **13**0.8, **13**0.3, **13**0.1, 128.5, 127.3, 127.1, 127.04, 126.98, 122.8, 122.7, 120.8, 111.4, 109.0, 95.8, 85.6, 44.5, 12.7 ppm (21 out of 22 signals expected); HRMS: m*/z *calcd. for C_26_H_24_N^+^: 350.1909; found: 350.1909 [M + H]+.

### 2.5. General procedure D: synthesis of 13, 15, and 17

A solution of the diethylaniline-substituted alkyne (0.**15** mmol, 1 equiv.) and TCNQ (0.**15** mmol, 1 equiv.) in 1,2-dichloroethane (20 mL) was stirred at 25 °C until complete consumption of starting material. After evaporation of the solvent, target chromophores **13**, **15**, and **17 **were isolated in 65%–83% yields by performing column chromatography (CC) (SiO_2_; DCM).

**Compound 13: **A dark-green solid; 327 mg, 65%; CC: (SiO_2_; DCM); m.p. **16**5–**16**7 °C; R*_f_* = 0.27 (SiO_2_; CHCl_3_); ^1^H NMR (400 MHz, CDCl_3_, 298 K): δ = 1.23 (t, *J* = 7.1 Hz, 6 H), 3.45 (q, *J* = 7.1 Hz , 4 H), 6.69 (d, *J* = 9.1 Hz , 2 H), 7.03 (dd, *J* = 9.5, 1.9 Hz, 1 H), 7.**14** (dd, *J* = 9.5, 1.9 Hz, 1 H), 7.25–7.35 (m, 3 H), 7.58 (t, *J* = 7.7 Hz , 2 H), 7.64 (t, *J* = 7.0 Hz , 1 H), 7.72 (dd, *J* = 8.7, 1.9 Hz, 1 H), 7.84–7.95 (m, 3 H), 8.**16** (s, 1 H); **^13^** C NMR (100 MHz, CDCl_3_, 298 K): δ = **17**2.9, **15**4.3, **15**2.1, **15**1.3, **13**5.9, **13**5.3, **13**5.1, **13**4.5, **13**2.7, **13**2.3, **13**1.8, **13**1.5, 129.8, 129.7, 128.1, 127.8, 125.3, 124.9, 124.7, 123.4, 1**15**.2, 1**15**.0, 1**13**.4, 1**13**.3, 112.6, 112.5, 87.3, 70.9, 45.1, 12.7 ppm; HRMS: m*/z *calcd for C_34_H_26_N_5_^+^: 504.2**18**8; found: 504.2192 [M + H]+.

**Compound 15: **A dark-green solid; 4**18** mg, 83%; CC: (SiO_2_; DCM); m.p. **17**4–**17**6 °C; R*_f_* = 0.23 (SiO_2_; CHCl_3_); ^1^H NMR (400 MHz, CDCl_3_, 298 K): δ = 1.24 (t, *J* = 7.1 Hz, 6 H), 3.46 (q, *J* = 7.1 Hz, 4 H), 6.67 (d, *J* = 9.1 Hz, 2 H), 7.**13** (d, *J* = 10.7 Hz, 1 H), 7.**17**–7.35 (m, 4 H), 7.40 (d, *J* = 9.5 Hz, 1 H), 7.54 (t, *J* = 7.7 Hz, 1 H), 7.59–7.66 (m, 2 H), 7.69 (t, *J* = 7.7 Hz, 1 H), 7.96 (d, *J* = 8.0 Hz, 1 H), 8.04 ppm (d, *J* = 8.3 Hz, 2 H); **^13^** C NMR (100 MHz, CDCl_3_, 298 K): δ = **17**2.4, **15**3.9, **15**3.2, **15**1.3, **13**6.8, **13**5.5, **13**4.4, **13**3.92, **13**3.90, **13**3.8, **13**0.4, 129.7, 129.6, 128.6, 127.3, 125.2, 125.1, 124.5, 124.0, 1**15**.1, 1**13**.1, 112.4, 112.2, 91.9, 71.1, 45.0, 12.7 ppm (27 out of 30 signals expected); HRMS: m*/z *calcd for C_34_H_26_N_5_^+^: 504.2**18**8; found: 504.2**18**8 [M + H]+.

**Compound 17: **A dark-green solid; 449 mg, 81%; CC: (SiO_2_; DCM); m.p. 191–193 °C; R*_f_* = 0.20 (SiO_2_; CHCl_3_); ^1^H NMR (400 MHz, CDCl_3_, 298 K): δ = 1.20 (t, *J* = 7.1 Hz, 6 H), 3.42 (q, *J* = 7.1 Hz, 4 H), 6.64 (d, *J* = 9.1 Hz, 2 H), 7.**13** (d, *J* = 10.4 Hz, 1 H), 7.22–7.36 (m, 4 H), 7.40 (dd, *J* = 9.5, 1.6 Hz, 1 H), 7.62–7.84 (m, 4 H), 7.87 (s, 1 H), 7.92 (d, *J* = 7.9 Hz, 1 H), 8.09 (d, *J* = 8.1 Hz, 1 H), 8.68 (d, *J* = 8.3 Hz, 1 H), 8.76 ppm (d, *J* = 7.8 Hz, 1 H); **^13^** C NMR (100 MHz, CDCl_3_, 298 K): δ = **17**2.3, **15**3.9, **15**3.0, **15**1.2, **13**6.8, **13**5.6, **13**4.4, **13**4.3, **13**3.4, **13**2.9, **13**2.1, **13**1.0, **13**0.3, **13**0.0, 128.2, 128.0, 127.82, 127.80, 125.5, 125.4, 125.1, 124.7, 124.1, 123.0, 1**15**.0, 1**14**.9, 1**13**.2, 112.5, 112.2, 92.1, 72.0, 45.1, 12.7 ppm (33 out of 34 signals expected); HRMS: m*/z *calcd for C_38_H_28_N_5_^+^: 554.2345; found: 554.2344 [M + H]+.

### 2.6. General procedure E: synthesis of 14, 16, and 18

A solution of the diethylaniline-substituted alkyne (0.**15** mmol, 1 equiv.) and TCNE (0.**15** mmol, 1 equiv.) in 1,2-dichloroethane (20 mL) was stirred at 25 ^o^C until complete consumption of starting material. After evaporation of the solvent, target chromophores** 14**, **16**, and **18 **were isolated in 95%–97% yields by performing column chromatography (CC) (SiO_2_; DCM).

**Compound 14: **A dark-purple solid; 4**15** mg, 97%; CC: (SiO_2_; DCM); m.p. 196–198 °C; R*_f_* = 0.27 (SiO_2_; CHCl_3_); ^1^H NMR (400 MHz, CDCl_3_, 298 K): δ = 1.26 (t, *J* = 7.1 Hz, 6 H), 3.49 (q, *J* = 7.1 Hz, 4 H), 6.71 (d, *J* = 9.5 Hz, 2 H), 7.60 (t, *J* = 7.5 Hz, 1 H), 7.68 (t, *J* = 7.5 Hz, 1 H), 7.80–8.00 (m, 6 H), 8.23 (s, 1 H); **^13^** C NMR (100 MHz, CDCl_3_, 298 K): δ = **16**9.3, **16**3.0, **15**2.8, **13**5.7, **13**3.1, **13**2.7, **13**2.0, **13**0.1, 129.93, 129.92, 129.4, 128.9, 128.1, 127.9, 124.3, 1**17**.7, 1**14**.8, 1**13**.9, 112.6, 112.2, 111.7, 86.6, 45.3, 12.6 ppm; HRMS: m*/z *calcd for C_28_H_22_N_5_^+^: 428.**18**75; found: 428.**18**75 [M + H]+.

**Compound 16: **A dark-purple solid; 4**15** mg, 97%; CC: (SiO_2_; DCM); m.p. 204–206 °C; R*_f_* = 0.30 (SiO_2_; CHCl_3_); ^1^H NMR (400 MHz, CDCl_3_, 298 K): δ = 1.25 (t, *J* = 7.1 Hz, 6 H), 3.48 (q, *J* = 7.1 Hz, 4 H), 6.71 (d, *J* = 9.4 Hz, 2 H), 7.50–7.62 (m, 2 H), 7.65 (t, *J* = 8.0 Hz, 1 H), 7.70–7.86 (m, 3 H), 7.98 (d, *J* = 8.0 Hz, 1 H), 8.07 (d, *J* = 8.7 Hz, 1 H), 8.10 ppm (d, *J* = 8.0 Hz, 1 H); **^13^** C NMR (100 MHz, CDCl_3_, 298 K): δ = **16**8.5, **16**4.1, **15**2.7, **13**4.8, **13**4.0, **13**3.0, **13**0.4, 129.9, 129.7, 129.3, 128.6, 127.6, 124.92, 124.85, 1**18**.4, 1**14**.8, 1**14**.0, 112.1, 111.8, 111.5, 91.9, 75.1, 45.1, 12.6 ppm; HRMS: m*/z *calcd for C_28_H_22_N_5_^+^: 428.**18**75; found: 428.**18**76 [M + H]+.

**Compound 18: **A dark-purple solid; 454 mg, 95%; CC: (SiO_2_; DCM); m.p.**15**7–**15**9 °C; R*_f_* = 0.33 (SiO_2_; CHCl_3_); ^1^H NMR (400 MHz, CDCl_3_, 298 K): δ = 1.24 (t, *J* = 7.1 Hz, 6 H), 3.47 (q, *J* = 7.1 Hz, 4 H), 6.71 (d, *J* = 9.3 Hz, 2 H), 7.67 (t, *J* = 7.5 Hz, 1 H), 7.75–7.85 (m, 5 H), 7.90, (s, 1 H), 7.94 (d, *J* = 7.9 Hz, 1 H), 8.12–8.**18** (m, 1 H), 8.71 (d, *J* = 8.4 Hz, 1 H), 8.75–8.85 (m, 1 H); **^13^** C NMR (100 MHz, CDCl_3_, 298 K): δ = **16**8.5, **16**4.3, **15**2.7, **13**3.3, **13**2.9, **13**2.6, **13**1.1, **13**0.7, **13**0.5, **13**0.1, 129.7, 128.5, 127.88, 127.87, 127.1, 126.0, 124.0, 123.0, 119.0, 1**14**.9, 1**14**.0, 112.1, 111.9, 111.6, 92.2, 75.9, 45.2, 12.7 ppm; HRMS: m*/z *calcd for C_32_H_24_N_5_^+^: 478.2032; found: 478.2032 [M + H]+.

## 3. Results and discussion

### 3.1. Synthesis of donor-substituted substrates 

**Scheme 1:**
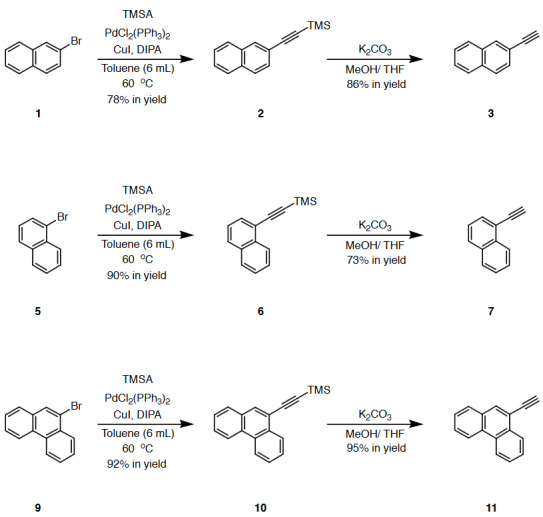


It was the initial goal of the project to prepare PAH-substituted alkynes by considering several key points such as solubility of the substrates, sufficient donor-activation for the subsequent CA-RE transformations, and commercial availability of the reagents. The reaction sequence to access substrates 4, 8, and 12, required for the synthesis of target push–pull structures initiated with the conversion of the aryl bromides 1, 5, and 9 to TMS-protected alkynes 2, 6, and 10 via Sonogashira cross-coupling reactions (Scheme 1). All three compounds 2, 6, and 10 were successfully synthesized according to the literature procedure reported by Ouyang and co-workers [39]. Upon subsequent deprotection using K_2_CO_3_ in MeOH/THF mixture, aryl alkynes 3, 7, and 11 were obtained in 86, 73, and 93 yields, respectively [39]. The final Sonogashira cross-coupling of PAH-substituted alkynes 3, 7, and 11 and *N*,*N*-diethyl-4-iodoaniline provided target donor-substituted substrates 4, 8, and 12 in 71, 90, and 64 yields, respectively. The reported synthesis could also be carried out between aryl halides 1, 5, 9, and *N*,*N*-diethyl-4-ethynylaniline. However, the stability of all three PAH-substituted alkynes under ambient conditions, in contrast to *N*,*N*-diethyl-4-ethynylaniline, guided us to follow the proposed synthetic strategy.

**Scheme 2:**
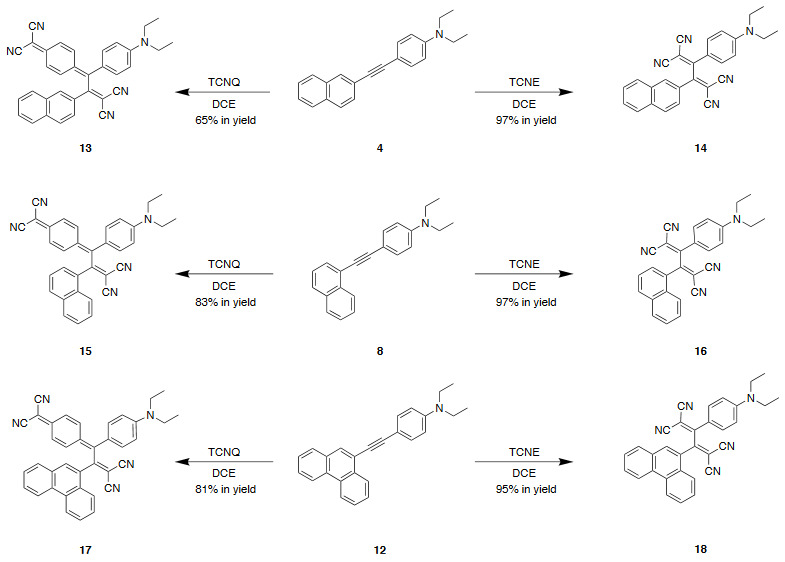


PAH (naphthene and phenanthrene) and donor group (diethylaniline)-substituted acetylenes 4, 8, and 12 were treated with strong acceptors TCNE/TCNQ for the final [2+2] CA-RE step under ambient conditions, which successfully afforded target push-pull chromophores in high yields. Even with the bulky TCNQ, reactions proceeded smoothly at room temperature (Scheme 2). No significant difference was observed regarding the effect of substituent positions on isolated yields. The yields were slightly lower in CA-RE transformations of TCNQ compared to that of TCNE, mainly due to solubility and purification difficulties that were encountered during column chromatography.

**Scheme 3:**
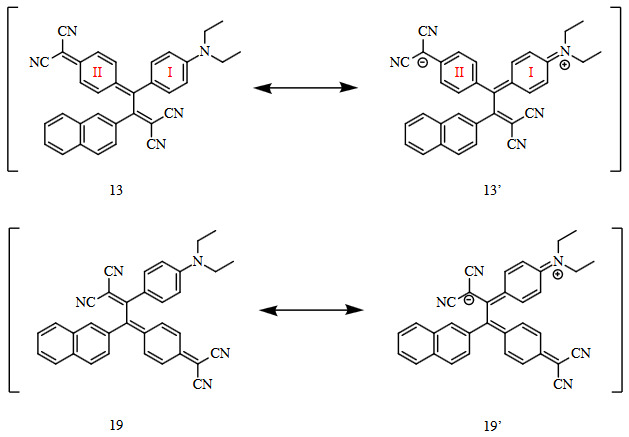


Although there are two possible regioisomers 13 and 19 that can be formed theoretically during the reaction of TCNQ and alkyne 4, the reaction was fully regioselective and afforded only 13 (Scheme 3). The reason behind this well-studied selectivity can easily be deducted from resonance structures of 13 and 19. Intramolecular charge transfer breaks the aromaticity of the diethylaniline ring (I) while forming a new one (II), as in the case of 13 [1,26].

### 3.2. UV/vis absorption spectra

**Figure 1:**
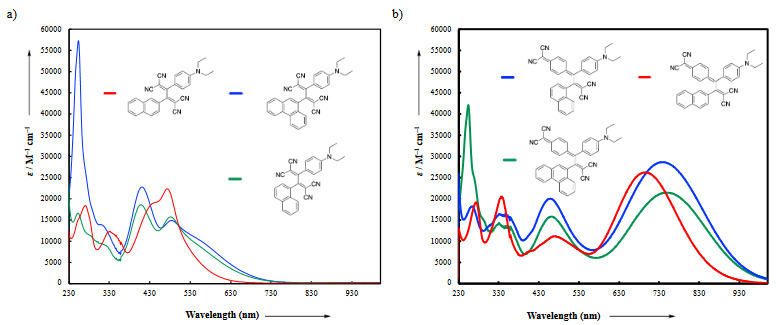
UV/vis spectra (CH2Cl2, 25 oC) of chromophores a) 14, 16, 18 and b) 13, 15, 17.

The formal [2+2] CA-RE of 4, 8, 12 with TCNE/TCNQ provided dark green and dark orange chromophores, respectively. The UV/vis absorption data was in full agreement with this observation (Figure 1a and b). While dark orange-colored compounds **14**, **16**, **18** possess intramolecular charge transfer (ICT) bands with λ_max_ values between 408 and 475 nm, ICT bands of dark-green chromophores **13**, **15**, **17** are significantly shifted to longer wavelengths and are located between 695 and 749 nm. This substantial difference in λ_max_ values can be explained by the extended π-conjugation pathways in compounds **13**, **15**, **17**, which decreases HOMO-LUMO band gaps [40]. The introduction of different PAH (phenanthrene, 1-naphthyl, and 2-naphthyl) substituents in **14**, **16**, **18 **leads to important differences in λ_max _values. 1-naphthyl and phenanthrene-substituted chromophores** 16** and **18** display a similar ICT bands at 408 and 411 nm, respectively (ε = **18**600 M^–1^ cm^–1^ for **16**;* ε* = 22,800 M^–1^ cm^–1^ for **18**). On the other hand, 2-naphthyl-substituted product **14** showed a bathochromically shifted band (λ_max_ *=* 474 nm, ε = 22,300 M^–1^ cm^–1^). The opposite trend was observed for the chromophores** 13**, **15**, **17. **Compounds **15** and **17** have ICT bands at 739 and 749 nm, respectively (ε = 28,700 M^–1^ cm^–1^ for **15**;* ε* = 21,500 M^–1^ cm^–1^ for **17**). 2-naphthyl-substituted product **13** exhibited hypsochromically shifted ICT band (λ_max_ *=* 695 nm, ε = 26200 M^–1^ cm^–1^). As it will be discussed in more detail in the theoretical section, the trend in λ_max _values is affected by PAH substituents sterically, not electronically. The steric restrictions from the substitution pattern of the PAHs **15** and **17** lead to a substantial deconjugation, as indicated by the large dihedral angle between the acceptor and the donor groups (**15**: 33 ° and **17**: 34 °). The dihedral angle in chromophore **13** was relatively smaller (28 °) compared to that of **15** and **17**. The large dihedral angle between the diethyl aniline donor and the cyano-based acceptor moiety prevents efficient linear π-conjugation in **15** and **17**, which leads to a bathochromic shift [41,42]. A similar but completely opposite trend was observed for the compounds **14** (**18** ^o^), **16** (31 ^o^), and **18** (33.2 ^o^). Despite the hypsochromic shift seen in λ_max _values, chromophores **16** and **18** also possess CT bands at around 474 nm with relatively smaller extinction coefficients compared to that of **14**. The decrease in these extinction coefficients can simply be explained by the observed deviation from planarity due to increase in dihedral angles of compounds **16** and **18**.

**Figure 2:**
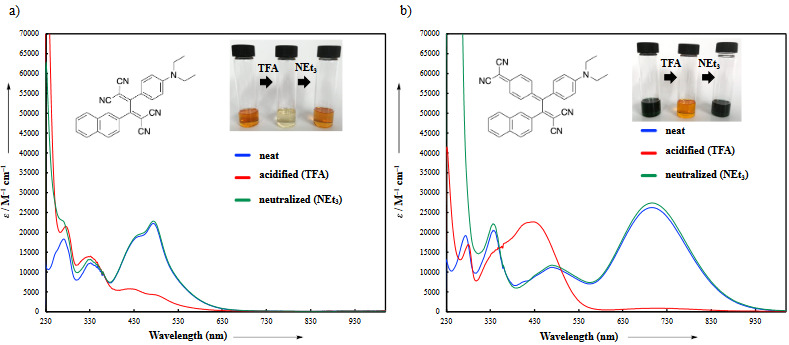
UV/vis spectrum of selected chromophores a) 14 and b) 13. The charge transfer band disappeared when acidifying the solution with trifluoroacetic acid and reappeared when neutralizing the solution with triethylamine.

All push-pull systems 13–18 feature strong CT bands that are assigned to intramolecular charge transfer from the donor diethylaniline group to acceptor polycyano units accessed by the CA–RE of TCNE and TCNQ. To further support this claim, protonation-reneutralization experiments were conducted by using CF_3_COOH (TFA) and NEt_3 _(Figure 2a and b). Treatment of selected chromophores with TFA resulted in the disappearance of the CT bands via protonation of the diethylaniline unit. Upon treatment with NEt_3_, re-neutralization occurred, and CT bands were successfully recovered. With these experiments, the CT nature of the low energy bands has been confirmed [40,43,44]. 

**Figure 3:**
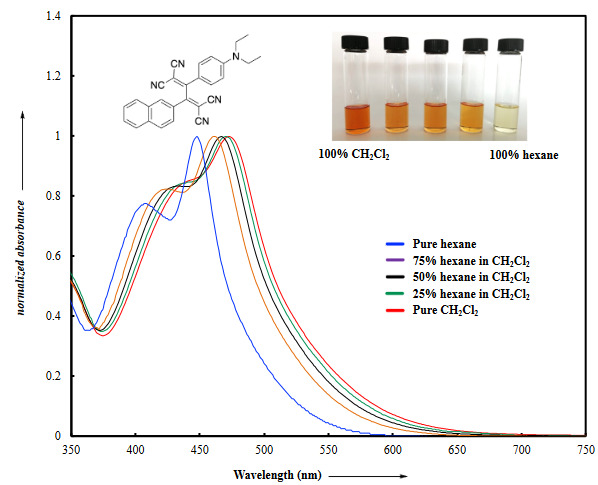
UV/vis spectra of chromophore 14 in CH2Cl2/hexane mixtures at 25 oC

**Figure 4:**
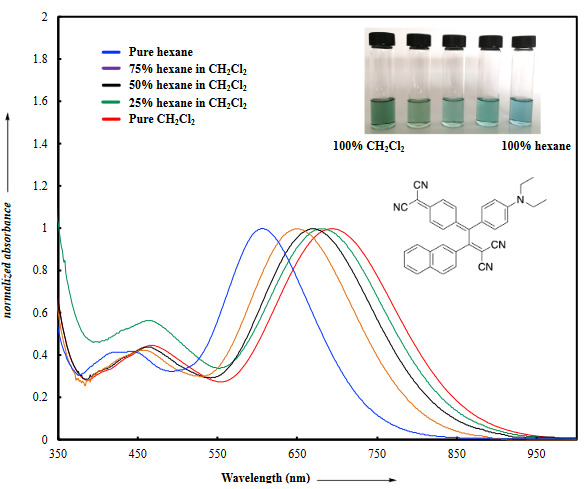
UV/vis spectra of chromophore 13 in CH2Cl2/hexane mixtures at 25 oC.

All push-pull systems 13–18 feature strong CT bands that are assigned to intramolecular charge transfer from the donor diethylaniline group to acceptor polycyano units accessed by the CA–RE of TCNE and TCNQ. To further support this claim, protonation-reneutralization experiments were conducted by using CF_3_COOH (TFA) and NEt_3 _(Figure 2a and b). Treatment of selected chromophores with TFA resulted in the disappearance of the CT bands via protonation of the diethylaniline unit. Upon treatment with NEt_3_, re-neutralization occurred, and CT bands were successfully recovered. With these experiments, the CT nature of the low energy bands has been confirmed [40,43,44]. 

### 3.3. Theoretical studies

All calculations (DFT and TD-DFT) were performed using Gaussian 09 program package [45]. The effect of different functionals and basis sets on chromophore **14 **has been evaluated. Since optimization results were obtained for B3LYP/6-31G(d), B3LYP/6-31++G(d,p), and CAM-B3LYP/6-31G(d), CAM-B3LYP/6-31++G(d,p) were very similar; further optimizations were obtained at the functional using basis set B3LYP/6-31G(d). As a solvation model, a conductor-like polarizable continuum model (CPCM) in CH_2_Cl_2_ was utilized. Initially, the highest occupied molecular orbital (HOMO) – lowest unoccupied molecular orbital (LUMO) and electrostatic potential (ESP) map analyses were used to explain ICT behavior in push-pull systems **13**–**18**. As can be seen in Table 1, HOMO is mainly located on the diethylaniline region of the molecules in all chromophores **13**–**18**. On the other hand, LUMO covers the area where cyano groups are present. Together with the partial overlap of HOMO and LUMO frontier orbitals, CT from diethylaniline groups to cyano groups can be claimed for the designed push-pull systems. In addition to FMO analysis, electrostatic potential maps were also analyzed to further evaluate CT interactions in push–pull chromophores. ESP representations describe the total charge density and the molecular polarity in push–pull systems [46]. Red and blue color codes have been used to identify the most negative and positive areas, respectively. As expected, red regions were located around cyano groups; on the other hand, blue regions were identified around the donor diethylaniline donor group.

**Table 1:** Molecular structures, HOMO and LUMO depictions, and ESP maps of 13–18. While the most negative areas are represented by red, the most positive areas are represented by blue color. ESP is mapped over the range –0.03 a.u (red). to 0.03 a.u (blue). B3LYP/6-31G(d) level of theory was used for DFT calculations.

	13	14	15	16	17	18
Molecular Structure	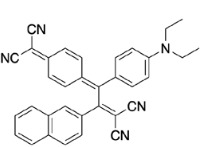	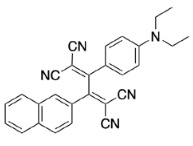	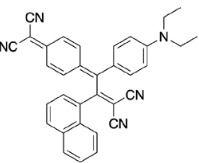	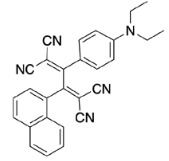	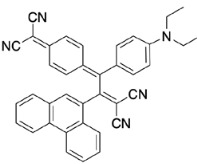	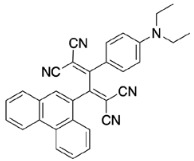
HOMO	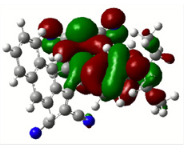	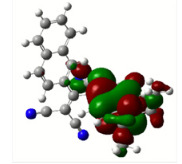	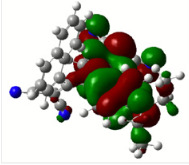	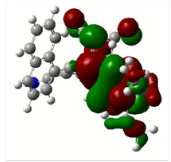	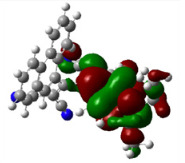	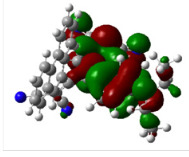
LUMO	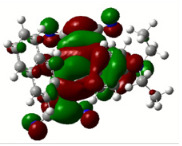	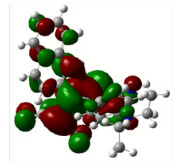	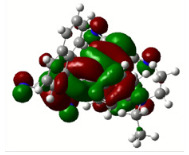	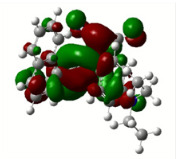	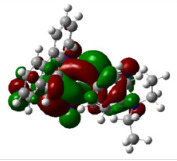	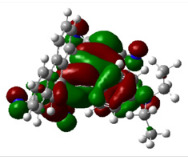
ESP	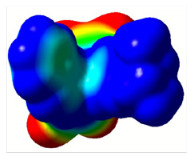	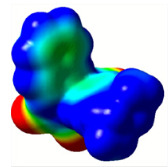	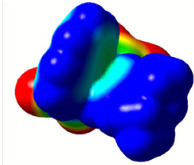	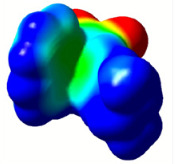	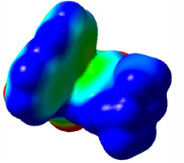	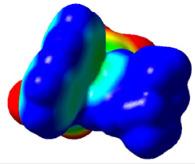

**Table 2:** Calculated quinoidal character values (δr) and total average atomic charges (δ) by ESP fitting on donor, acceptor and PAH parts of push–pull chromophores 13–18.

Compound	(δr) (Å)	δ donor (eV)	δ acceptor (eV)	δ PAH (eV)
13	Ring I = 0.049, Ring II = 0.072	0.487	–0.614	0.124
14	Ring III = 0.048	0.388	–0.573	0.188
15	Ring I = 0.048, Ring II = 0.071	0.463	–0.476	0.012
16	Ring III =0.045	0.362	–0.456	0.094
17	Ring I = 0.047, Ring II = 0.071	0.494	–0.434	–0.071
18	Ring III = 0.045	0.360	–0.452	0.094

**Figure 5:**
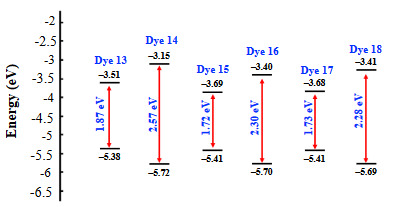
Energy level diagram of the frontier orbitals of push–pull dyes estimated by DFT calculations.

The energy level diagrams of push–pull dyes are depicted in Figure 5. The estimated frontier orbital energy levels showed that there is a substantial increase in the HOMO–LUMO band-gap of TCNE adducts **14**, **16**, and **18** compared to TCNQ adducts** 13**, **15**, and **17 **due to the shortened π-conjugation pathway in the former case. The theoretical band gaps for push–pull dyes are 1.87, 2.57, 1.72, 2.30, 1.73, 2.28 eV, respectively. The theoretically obtained band gap values are slightly higher compared to the optical band gaps 1.44, 2.28, 1.31, 1.82, 1.31 1.77 eV [47]. No significant improvement in band gaps were obtained from the TD-DFT band gaps 2.01, 2.77, 1.88, 2.52, 1.89, 2.51 eV for compounds **13**, **14**, **15**, **16**, **17**, and **18** respectively. However, the trend in theoretical band-gap values can also be seen in experimental band-gap values for both TCNE and TCNQ adducts. *E*_red,1 _values were reported for the benchmark cyano acceptors, such as TCNE (–0.32 V), and TCNQ (–0.25 V). A key property of this class of compounds is their electron-accepting power increasing from TCNE to TCNQ. It is also well-known that CA-RE products from TCNQ possess smaller band-gap compared to the products from TCNE. This can also be explained by the increased conjugation length in between donor and acceptor cyano groups in TCNQ products [1].

**Figure 6:**
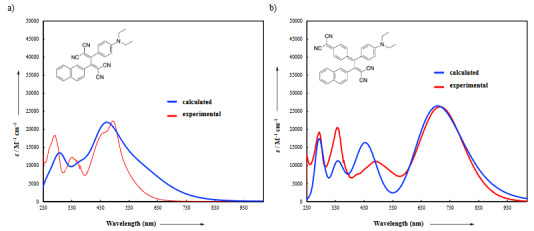
a) Calculated (red-shifted by 0.6 eV, scaled by 1.87, red line) TD-DFT:CAM-B3LYP/6–31G(d) level of theory in CH2Cl2 and experimental UV/vis spectrum of 14 in CH2Cl2 (blue line). b) Calculated (red-shifted by 0.2 eV, scaled by 2.29, red line) TD-DFT:CAM-B3LYP/6–31G(d) level of theory in CH2Cl2 and experimental UV/vis spectrum of 13 in CH2Cl2 (blue line).

Figure 6 shows both theoretical and experimental UV/vis spectra for the selected compounds **13** and **14**. Time-dependent density theory calculations (CAM-B3LYP/6-31G(d)) were applied on optimized push-pull structures of **13**–**18** with CPCM solvation in CH_2_Cl_2_. In order to match the theoretical UV/vis spectra with the corresponding theoretical spectra, wavelengths were red-shifted by 0.2 eV and 0.6 eV for compounds **13** and **14**, respectively. Scaling of extinction coefficients was also required (scaled by 2.29 for **13** and by 1.87 for **14**) since these values are slightly overestimated by TD-DFT calculations. However, the overall shapes of the theoretical spectra are relatively well-estimated (see Figures 6a and b). Although the reason for the overestimation in molar absorption coefficients in TD-DFT calculations is still unclear, the choice of basis set and solvation method may be responsible from a slight inaccuracy in dihedral angles that may facilitate donor-acceptor conjugation efficiency and increase the molar absorption coefficients. All push–pull chromophores **13**–**18** possess charge-transfer bands originated from HOMO to LUMO transitions. The observed error in the calculated transitions are in the expected range for similar push–pull systems reported in the literature [48].

Quinoid character values (d*r*) are commonly used to predict the amount of charge transferred in push-pull systems [11,19]. If there is no charge-transfer, d*r *value equals 0 and represent a perfect benzene structure. On the other hand, fully quinoidal structures possess d*r *values in between 0.08–0.1. Bond lengths from optimized geometries of compounds **13**–**18** have been used for the d*r *value calculations (Table 2). A substantial quinoid character values (d*r* = 0.045–0.049) were predicted for diethylaniline donor groups in all chromophores. A slight increase in d*r *values of TCNQ adducts was observed compared to TCNE adducts. The predicted quinoid characters were comparable to earlier reports on CA-RE products [11,19]. Inspired by the literature on strong D-A systems [49,50] these results were further confirmed by total average atomic charges (δ) by ESP fitting on donor, acceptor and PAH parts of push–pull chromophores. Atomic charges were calculated based on the ESP fitting scheme of Merz–Singh–Kollman (MK) [51]. All chromophores were divided into three parts: diethylaniline donor, PAH substituents, and cyano-based acceptor groups (Table 2). Accordingly, all chromophores possess charge-transfer from donor to acceptor units. The total average charge differences between donor and acceptor units are significantly higher in quinone based structures **13**, **15**, and **17**. The most enhanced charge-transfer capacity were predicted for compound **13** with δ donor = 0.487 eV and δ acceptor = –0.6**14** eV. This result is fully consistent with the highest quinoidal character value calculated for chromophore **13** (d*r* = 0.049). In summary, chromophores obtained by [2+2] CA–RE of TCNQ are better chromophores in terms of charge-transfer interactions compared to chromophores obtained by [2+2] CA–RE of TCNE. Additionally, chromophores-substituted with 2-naphthyl group enhance donor-acceptor properties compared to chromophores substituted with phenanthrene and 1-naphthyl. Presumably, deviation from planarity is the main reason behind this observation due to bulky phenantrene and geometrically constrained 1-naphthyl substitutents. 

**Table 3:** The electric dipole moment μ (D), EHOMO, ELUMO, ∆E (EHOMO–ELUMO), electronegativity (χ), global chemical hardness (η), global softness (σ), average polarizability [α(tot)], first hyperpolarizability [β(tot)] at the CAM-B3LYP/6-31++G(d,p) level of theory in CH2Cl2.

	13	14	15	16	17	18
μ (D)	27.9240	19.8620	27.8594	19.9083	26.5140	20.3709
EHOMO (eV)	–5.38	–5.72	–5.41	–5.70	–5.41	–5.69
ELUMO (eV)	–3.51	–3.15	–3.69	–3.40	–3.68	–3.41
∆E (eV)	1.87	2.57	1.72	2.30	1.73	2.28
χ (eV)	4.445	4.435	4.550	4.550	4.545	4.550
η (eV)	0.935	1.285	0.860	1.150	0.865	1.140
σ (eV–)	1.0695	0.7782	1.1627	0.8695	1.1560	0.8771
α(tot) (×10–24 esu)	135.891	85.746	136.768	83.060	148.505	94.295
β(tot) (×10–30 esu)	451.403	198.435	536.872	228.638	537.842	239.935

The successful utilization of organic molecules in nonlinear optics motivates researchers to develop rational design strategies [52,53]. The fast and inexpensive theoretical calculations compared to experimental measurements provide a great advantage for the design of NLOphores with tailor-made properties. The most common NLOphore structures are generally containing D-π-A-type molecular frameworks [54,55]. Strong charge-transfer interactions in push-pull systems **13**–**18** motivated us to evaluate NLO properties using computational tools. To predict the NLO properties, DFT calculations have been applied on compounds** 13**–**18** using basis set CAM-B3LYP/6-31++G(d,p) level of theory. The electronegativity (χ), global chemical hardness (η), global softness (σ), electric dipole moment μ (D), average polarizability [α_(tot)_], and first hyperpolarizability [β_(tot)_] values were calculated according to the equations (1–6) shown below. 

β = [(βxxx + βxyy + βxzz)^2^ + (βyyy + βxxy + βyzz)^2^ + (βzzz + βxxz + βyyz)2]^1/2^ (1)

α = 1/3 (α*xx*+ α*yy* + α*zz*) (2)

μ = [(μ_x_)^2^ + (μ_y_)^2^ +( μ_z_)^2^]^1/2^ (3)

χ = –1/2 (E_HOMO _+ E_LUMO_) (4)

η = –1/2 (E_HOMO_–E_LUMO_) (5)

σ = 1/ η (6)

Overall results are summarized in Table 3. Molecular geometry is crucial as it dictates the NLO properties of the compounds. There are several strategies to modulate NLO responses, such as changing solvent choice, altering donor-acceptor strength, or π-linker length. In this study, we mainly focused on the effect of PAH rings on average polarizability [α_(tot)_] and first hyperpolarizability [β_(tot)_] values. When we compare TCNE and TCNQ adducts, the lowest [α_(tot)_] and [β_(tot)_] values were predicted for chromophores **13** and **14**, which possess naphthalene groups substituted at two positions. For the rest of the compounds, differences in [α_(tot)_] and [β_(tot)_] values are not very significant. Compounds with the bulky phenanthrene or naphthene group substituted at one position exhibited almost similar NLO responses. This prediction can be supported by the calculated and optically measured band gaps for **13** and **14**. The HOMO-LUMO energy gap for **13** is significantly larger compared to **15** and **17**. A similar trend can also be seen in TCNQ adducts. We found a general trend by which β_(tot)_ increases with the size of the spacer between donor and acceptor groups as can be seen by higher β_(tot)_ values in TCNQ products compared to those of TCNE products. We presume that smaller band-gap results in more efficient charge transfer and, as a result, larger NLO responses, as can be seen in Table 2. Optimized structures also displayed significant deviation from planarity in the case of compounds **15** (33 ^o^), **17** (34 ^o^), **16** (31 ^o^), **18** (33.2 ^o^) compared to **13** (28 ^o^), and **14** (**18** ^o^). Accordingly, we have shown that substituent groups can play an important role in modulating NLO properties of push-pull type chromophores, although they are mainly utilized as solubilizing groups or side groups to improve the physical properties of chromophores. The highest predicted β_(tot) _value in this study is 537.842 × 10^–30 ^esu for chromophore **17**. That value is 8**15**0 times larger than the benchmark NLO material urea, β_(tot) _value of 0.066 × 10^–30^ esu, calculated at the CAM-B3LYP/6-31++G(d,p) [22]. In the final part, the chemical properties of chromophores will be discussed using equations (4–6). The results are very promising when compared with the literature. For example, push-pull system, *p*-nitroaniline, possesses β_(tot) _value of 9.2 × 10^–30^ esu [56]. Similarly, [60] fullerene-fused dihydrocarboline derivative is calculated to have* β*_(tot) _value of 54 x 10^–30^ esu [57]. In another study, β_(tot) _values of 21 – 286 × 10^–30^ esu are reported for push-pull 1,3-thiazolium-5-thiolates [58].

Koopmans’ theorem states that HOMO and LUMO energies are related to ionization potential and electron affinity, respectively. Accordingly, the Mulliken electronegativity (χ) can be estimated by equation 4. Besides electronegativity, chemical hardness (η) is another term to be used for chemical behavior predictions of materials. Global hardness is directly related to the HOMO-LUMO gap and can be defined as the resistance of an atom to charge-transfer. Equations 4 and 5 show that larger HOMO-LUMO gaps are required to improve hardness values. Compounds **14**, **16**, **18** are predicted to have higher global hardness values with their larger band gaps compared to **13**, **15**, and **17**. An opposite trend can be seen in global softness (σ), values as expected from Equation 6. In summary, compounds **13**, **15**, and **17** are expected to be more reactive compared to** 14**, **16**, and **18**.

## 4. Conclusions

In this study, 6 new polycyclic aromatic hydrocarbon-substituted push–pull chromophores have been synthesized using click-type [2+2] cycloaddition-retroelectrocyclization. The synthetic approach worked smoothly under ambient laboratory conditions without requiring heat or catalyst. Optoelectronic properties of the highly-colored chromophores were investigated by using both experimental (UV/vis spectroscopy) and computational methods. Solvatochromism and pH studies were performed for all 6 chromophores. Computational studies (TD-DFT, electrostatic potential maps, HOMO-LUMO orbital depictions) were further confirmed that all chromophores undergo intramolecular charge transfer. The HOMO-LUMO energy gap of dye **13** is found to have the largest energy gap resulting in worse charge-transfer properties as compared to **15** and **17**. A similar trend was also observed for chromophores **14**, **16**, and **18**. PAH-substituted push-pull chromophores are predicted to have significant NLO properties, and these properties can simply be tuned by changing substituent PAH structures. The present study provides valuable knowledge for the design and synthesis of new NLOphore systems in the near future.
